# Free sugar intake and associated factors among Sri Lankan preschool children

**DOI:** 10.1186/s40795-022-00638-0

**Published:** 2022-11-21

**Authors:** Shanika Mututanthri, Tharanga Thoradeniya, Anil Samaranayake, Rebecca Harris

**Affiliations:** 1 Ministry of Health, No. 385, Rev. Baddegama Wimalawansa Thero Mawatha, Colombo, 10 Sri Lanka; 2 Department of Biochemistry and Molecular Biology, Faculty of Medicine, University of Colombo, Colombo, Sri Lanka; 3 Department of Public Health, Policy and Systems, University of Liverpool, Whelan Building, Quadrangle, Liverpool, L69 3GB UK

**Keywords:** Free sugar intake, Sugary foods and beverages, Risk factors, Sugar policy, Preschool children

## Abstract

**Background:**

Excessive free sugar intake tends to be associated with unpleasant health consequences, such as dental caries and unhealthy weight gain in children, as well as a number of noncommunicable diseases in adults. The WHO suggests that the best method for addressing these issues is to reduce free sugar consumption throughout life, in order to be successful, these measures should be implemented as early as possible. This makes the early formative years of preschool an important point for possible intervention. To confirm this, baseline information on current sugar intake levels is needed, as well as identification of factors associated with high levels of consumption.

**Methods:**

This cross-sectional study was conducted in the district of Colombo, Sri Lanka. The probability proportionate to size technique combined with cluster sampling was used to select a representative sample of 813 children aged 4-5-years from 82 preschools. We developed, and validated a quantitative food frequency questionnaire (FFQ) to assess free sugar consumption. Data on correlated factors were collected from caregivers using a pre-tested self-administered questionnaire.

**Results:**

Based on the data, the daily median (IQR) free sugar intake of preschool children was 57.9 (33.2-95.8) grams/day (approximately to 14.5 (8.3-23.9) teaspoons/day) or 21.1% (12.5-34.9%) of their daily energy requirements. The WHO recommends limiting sugar intake to less than 5% of total energy intake; however, the current level is fourfold, and 96% of children consume higher percentage of energy from free sugar than recommended. In terms of total daily sugar consumption, bakery products accounted for 27%, followed by biscuits (15%), and table sugar (15%). Increasing maternal education level significantly reduced sugar intake (*p* = 0.04). Children of other ethnicities ate more sugar than the Sinhala children (*p* = 0.01). There was higher sugar intake among those who ate while returning from preschool (*p* < 0.001), while watching television (*p* < 0.001), and those who had school-going siblings (*p* = 0.02).

**Conclusion:**

Among preschool children, free sugar consumption levels were very high and most of the children consumed more sugar than is recommended; which warranted urgent actions to curb sugar intake among them.

## Introduction

The modern food processing industry has introduced many unhealthy ingredients into the human diet, including a massive amount of free sugar, which presents an increasing challenge to public health, especially in developing countries [1]. A growing body of evidence suggests that excess free sugar plays a dominant role in causing a number of non-communicable diseases (NCD), including obesity [2, 3], diabetes [4, 5], cardiovascular diseases [6, 7], several forms of cancers [8, 9], and dental caries [10–12]. Although excess free sugar poses numerous health risks throughout our lives, dental caries and obesity have been identified as the primary consequences in childhood [13, 14]. In Sri Lanka, early childhood caries is a major health problem, affecting approximately 63% of five-year-old children [15]. Childhood obesity is also a growing health problem, particularly among adolescents [16]. Therefore, addressing rising rates of NCDs via sugar policy is a key concern, especially in developing countries that are disproportionately affected by this challenge [17]. Sri Lanka is one such context in which addressing the consumption of free sugars in the early years is an important issue that underpins efforts to prevent both dental caries and wider health impacts.

WHO guideline recommends maintaining free sugar intake below 10% of total energy intake throughout life, with the aim of reducing the risk of NCDs in both adults and children, focusing specifically on preventing and controlling unhealthy weight gain and dental caries [13]. This recommendation was based on moderate quality evidence from observational studies. However, because low quality evidence from ecological studies also exists showing a positive dose–response relationship between free sugars intake and disease, WHO have also issued a conditional recommendation that free sugars intake be further limited to less than 5% of total energy intake. For this purpose, the World Health Organization (WHO) defines free sugar as “all monosaccharides, and disaccharides added to foods and beverages by the manufacturer, cook or the consumer and sugars naturally present in honey, syrups, fruit juices and fruit juice concentrates” [13]. In this case, intrinsic sugars, which are present within the cell structures, were not included because of their low bioavailability. If Sri Lanka were to follow WHO recommendations, an essential first step would be to establish the current level of sugar intake in the country.

According to current understanding, taking a life-course approach is the best strategy to combat the onset of diet-related chronic diseases [18, 19]. Dietary habits in early life are crucial for determining eating behaviours in adolescence and adulthood [20]. Since preschool years mark a significant milestone in lifestyle development, shaping children ‘s eating habits at this early age is relatively easy [20, 21]. Accordingly, this study aimed to assess the intake of free sugar among preschool children aged four and five in order to plan interventions and preventive measures addressing early childhood caries, with the secondary goal of informing preventive interventions related to wider sugar-related diseases throughout their lifetime. The study had two objectives: first, to describe the level of free sugar intake in a key age group in Sri Lanka and second, to identify factors associated with excessive sugar intake. The second objective was to identify potential determinants of sugar intake habits as a prerequisite to applying health promotion principles aimed at achieving sustainable behavioural changes to curb excessive free sugar intake.

## Methods

This study was approved by the ethics review committee of the Faculty of Medicine, University of Colombo (EC-17-001), and written informed consent was obtained from all caregivers who participated in the study.

### Study population and sampling

A preschool-based cross-sectional study was conducted in Colombo District, Sri Lanka. This involved preschool children aged four to five years old (48 to 71 months). Sampling was approached as a two-stage technique: selecting a set of pre-schools within the Sri Lankan district involved in the study (Columbo) and then inviting all preschoolers and their carers within those preschools, to participate in the study. A sample size of 860 was calculated based on a formula for calculating population proportions [22] after adjusting for cluster sampling errors and possible non-response. A total of 82 clusters (preschools) were selected from 16 administrative divisions in the district, using a probability proportionate-to-size technique. The study inclusion criteria were 4-5-years-old pre-schoolers who had been living in the Colombo district from birth, whose primary caregiver was available, and who were not on a special dietary plan. Of the 860 invited, 813 dyads of children and their primary caregivers participated in the study (response rate, 94%).

### Study instruments

In the literature, several dietary assessment methods have been used to assess the relationship between diet and disease; among these, the food frequency questionnaire (FFQ) has several advantages. Firstly, the FFQ assesses the normal daily intake of particular foods or beverages [23, 24] excluding short-term variations related to seasonal changes, special occasions, or illnesses [23, 25]. Additionally, this is the only method that allows for evaluation of dietary data over an extended period [23, 26], which makes it helpful for exploring the relationship between sugar intake levels, dental caries status, and the general nutritional status of children. Furthermore, FFQs are relatively simple and easy to administer in large-scale community-based studies, with comparatively little burden on respondents [23].

We collected dietary data using a 67-item food frequency questionnaire (FFQ) and also a 24-hour dietary record (24hDR) which was completed at home for 3 days. 24hDr allows data to be collected in a prospective way and avoid issues related to recall bias. A food frequency questionnaire was developed for this study using data from 513 preschool children, and food items were selected that contributed 95% to the variation in free sugar intake. It was validated among 108 preschool children against a three-day dietary record and was found to be a valid and reliable tool for measuring free sugar intake. Based on a wide literature search, the researchers developed a questionnaire to assess factors that might be associated with free sugar intake in children. The questionnaire was pretested on 20 preschool children who lived outside the Colombo District.

### Data collection

From each preschool, data were collected from groups of between eight to twelve children. Trained interviewers visited the preschools and administered questionnaires to primary caregivers of the selected children. The investigators gave clear instructions on how to fill out the FFQ based on the child’s usual diet during the past 3 months followed by a practice session. The participants filled out the FFQs themselves while the investigators demonstrated the portion sizes in the PowerPoint presentation displayed on a laptop. After providing the necessary directions, the 24hDR was distributed for completion at home on one weekend day and two weekdays, which were collected later.

### Data analysis

For the purpose of the current study, the researchers compiled a comprehensive sugar composition database for all food and beverage items in the FFQ using standard methods [27, 28] because the available food composition databases for Sri Lanka do not provide the sugar content for most sugary foods in the FFQ.

Statistical analysis was performed using Statistical Package for the Social Sciences (SPSS) version 21.

FSI - Free Sugar Intake from a particular food/beverage item (g/day).

AI - Amount (g/ ml) of Intake.

FI - Frequency of Intake.

FSC -Free Sugar Concentration of the item (g/100 g or g/100 ml)

$$\textrm{FSI}=\textrm{AI}\ \textrm{x}\ \textrm{FI}\ \textrm{x}\ \textrm{FSC}$$

- ▪ Data obtained from the FFQ were used to calculate the amount of free sugar intake. This was calculated as follows:
  The daily intake of free sugar was calculated by dividing the weekly intake by seven and the monthly intake by 30.4. The total daily intake was calculated by adding the daily sugar intake values for each item.
- ▪ The three 24hDR were used to calculate the composite frequency of sugar intake. We counted the number of times a child consumed sugary items per day without considering how many items they combined at a time; this was called “*composite frequency”*. The average daily frequency was calculated. For instance, when a child consumed a biscuit, a piece of cake, and milk with sugar at tea time, the frequency was taken as one (as per the FFQ, this would be considered three times).
- ▪ In this study, the total energy intake of participants was used to calculate the percentage of energy intake from free sugar. As this was beyond the scope of this study, the energy requirement of the child was calculated based on age and body weight as a proxy measure. Approximately a 4-6-year-old child requires 82 kcal/kg/day [29]. The calculations were performed as follows:
  - EIFS - Energy Intake from free sugar as a percentage of the total energy requirement.
  - ASI - Amount (g) of sugar intake per day.
  - ERD - Total energy requirement per day (kcal/day)
    $$\textrm{EIFS}=\frac{\textrm{ASI}\ \textrm{x}\ 4\ast }{\textrm{ERD}}$$

*One gram of sugar provides 4 kcal of energy.

- ▪ To identify the factors associated with sugar intake levels, bivariate analysis was first performed, followed by multivariate analysis using multiple logistic regression models with backward elimination.

## Results

The study sample was comprised of 4-5-year-old preschool children with the majority (70%) being 4 years old (48-59 months). Most were of Sinhala ethnicity. An equal number of girls and boys participated in the study (Table 1).

**Table 1:** Socio-demographic characteristics of participants (*N* = 813)

Variables	Number	Percentage (%)
**Sex**		
Girls	407	50.1
Boys	406	49.9
**Age (months)**		
48- 59	567	69.7
60 - 71	246	30.3
**Ethnicity**		
Sinhala	672	82.7
Other	141	17.3
**Total**	**813**	**100**

### Amount of free sugar intake

As shown in Table 2, the mean (SD) and median (IQR) amount of free sugar intake were 79 (68.6) g/day and 57.9(33.2- 95.8) g/day (equivalent approximately to 14.5 (8.3-23.9) teaspoons/day), respectively. Free sugar contributed 21.1% (12.5-35.9%) of total energy intake on average. The median (IQR) composite frequency of sugar intake was four times (2.7-6.1) per day.

**Table 2:** The amount and of free sugar intake among the participants (*N* = 813)

	Sugar energy* as a percentage of total energy requirement**	Amount of sugar intake (g/day)	Frequency of sugar intake (times/ day)
Range	0.5-192.3	2.5 - 520.0	0 - 15.8
Mean (SD)	29.0 (25.3)	79.0 (68.6)	4.9 (3.2)
Median (IQR)***	21.1 (12.5 - 35.9)	57.9 (33.2 - 95.8)	4.0 (2.7 - 6.1)

*Sugar energy = amount of sugar (g) x 4 Kcal/ g (1 g of sugar provides 4kCal of energy)

**Total energy requirement = bodyweight x energy requirement/ kg body weight/ day

***IQR = Inter-Quartile Range

### The percentage contribution of different sugary food and beverage groups to total free sugar intake

**Fig. 1:**
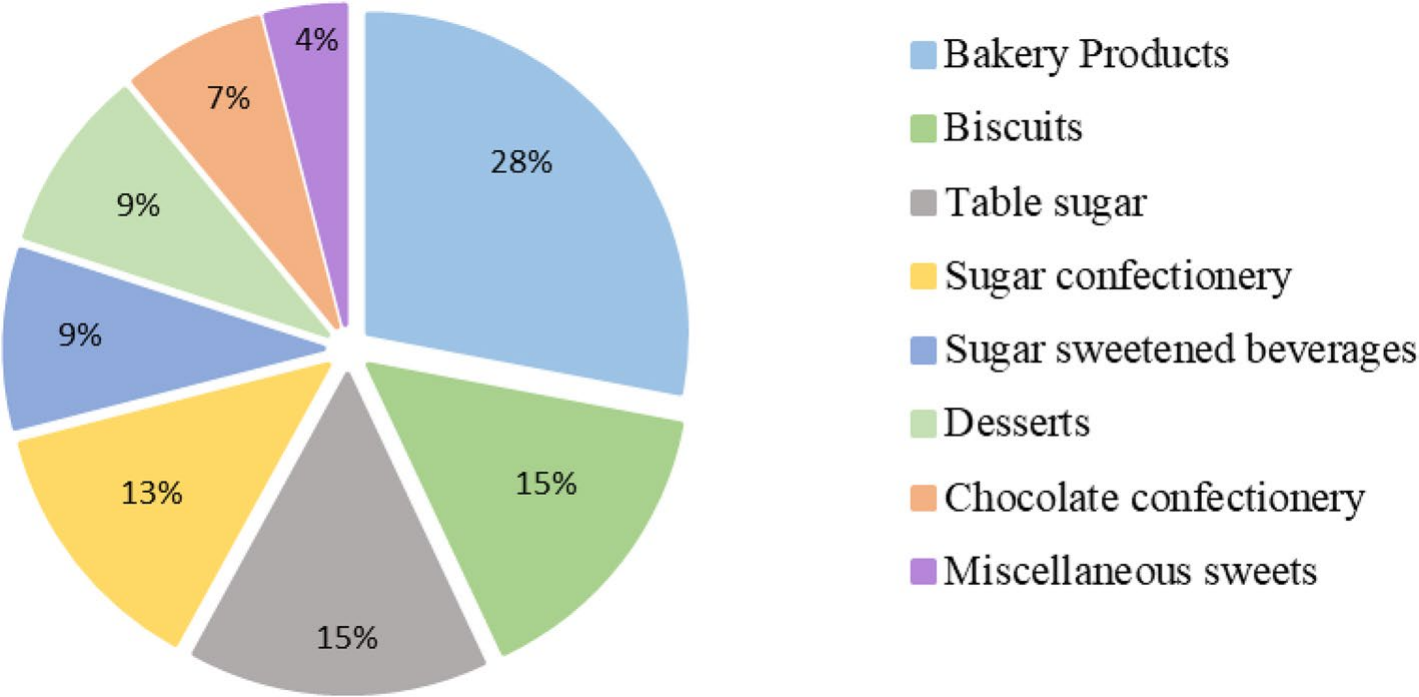
The percentage contribution of different sugary food and beverage groups to total free sugar intake

The contribution of each food and beverage group to total free sugar intake is shown in Fig. 1. To identify excess consumption, biscuits were analysed separately from the other bakery products.

The main contributors to the total intake of free sugar were bakery products (28%), followed by biscuits (15%), and table sugar (15%).

**Fig. 2:**
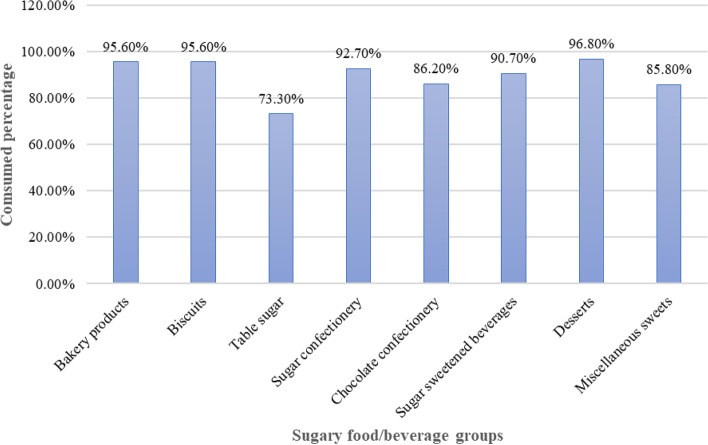
The percentage (number) of children who had consumed different sugary food and beverage groups

Figure 2 shows the percentage of participants who consumed each food category at least once a month.

The largest percentage of children consumed desserts (96.8%), followed by biscuits (95.6%) and bakery products (95.6%).

### Factors associated with the amount of free sugar intake

Multivariate logistic regression with backward elimination was performed after bivariate analysis to identify factors associated with high free sugar intake. As shown in Table 3, the final model retained six predictor variables.

**Table 3:** An analysis of multiple logistic regression models relating total free sugar intake to the factors associated with it dependent variable: Total free sugar intake

	Unstandardized Coefficients		Standardized Coefficients Beta	t	Sig. (p)	95% Confidence Interval for B	
	B	Std. Error				Lower Bound	Upper Bound
(Constant)	69.27	15.979		4.335	< 0.001	37.8	100.658
**1. Ethnicity**	17.27	6.882	0.09	2.51	0.012	3.7	30.8
Sinhala Other ethnic groups*							
**2. Maternal education level**	−9.82	4.792	−0.08	−2.04	0.041	−19.2	−0.4
Up to GCE O/L** bellow Up to GCE A/L*** above							
**3. School-going siblings**	10.39	4.754	0.08	2.18	0.029	1.1	19.7
No Yes							
**4. Eating while returning from preschool**	38.46	5.245	0.28	7.33	< 0.001	28.2	48.7
No Yes							
**5. Eating while watching TV**	47.47	7.269	0.25	6.53	< 0.001	33.2	61.7
No Yes							
**6. Dental clinic attendance**	−19.2	5.093	−0.14	−3.78	< 0.001	−29.3	−9.3
No Yes							

*Non-Sinhala- Tamil, Moor and others amalgamated

**GCE O/L- General Certificate of Education Ordinary Level (Grade 11)

**GCE A/L- General Certificate of Education Advanced Level (Grade 13)

R square = 0.215, Adjusted R Square = 0.207

The R^2^ value for the final model was 0.215, indicating a 21.5% variance in the total sugar intake. This model showed an F value of 25.662 with a statistically significant *p*-value (*p* < 0.01).

Maternal educational level and dental clinic attendance were significantly and negatively associated with free sugar intake. A child whose mother had obtained at least G.C.E. (advanced level) education was likely to consume significantly less sugar than a child whose mother had obtained up to G.C.E. (ordinary level) (*p* = 0.04). Children who visited a dental clinic at least once had a significantly lower sugar intake than those who had never visited a dental clinic (*p* < 0.001).

Ethnicity other than Sinhala (*p* = 0.01), the presence of school-going siblings (*p* = 0.02), eating habits such as eating while returning from preschool (*p* < 0.001), and eating while watching television (*p* < 0.001) were significantly associated with high intake levels of sugar.

As shown in Table 4, free sugar intake from particular groups of sugary foods was significantly related to the regular presence of the same group of foods at home.

**Table 4:** The relationship between free sugar intake from different groups of sugary foods and the presence of the same group of foods at home on a regular basis

	Number	Mean	Median	Significance
***Biscuits***		***Sugar from biscuits***		
Yes	698	12.1	5.8	*z* = 3.77
No	115	8.3	5.6	*p* < 0.001
***Sugar sweetened beverages***		***Sugar from sugar sweetened beverages***		
Yes	182	8.3	3.7	*z* = 3.55
No	631	5.6	2.3	*p* < 0.001
***Chocolate confectionery***		***Sugar from chocolate confectionery***		
Yes	201	6.1	2.7	*z* = 3.77
No	612	4.3	1.4	*p* < 0.001
***Fruits***		***Total free sugar***		
Yes	703	73.9	53.2	*z* = 1.99
No	110	83.3	66.3	*p* = 0.04
**Total**	**813**			

Among those who regularly have biscuits (*p* < 0.001), beverages (*p* < 0.001), and chocolates (*p* < 0.001) at home, sugar intake from relevant items was significantly higher than that of their counterparts. The opposite pattern was observed among children for whom fruits were available at home on a regular basis, who had a significantly lower intake of total free sugar than those who did not (*p* = 0.04).

## Discussion

Among many other factors, assessing dietary intake is critical for understanding how nutrition plays a role in preventing or promoting diseases. This paper discusses the extremely high intake of free sugar among preschool children in Sri Lanka along with possible associated factors. The limitations of the study include the intrinsic limitations of the methods of dietary assessment used since the FFQ rely on memory and are therefore susceptible to overestimations and underestimations. There may be some responder bias in reporting lower levels of sugar consumption because of the fear of being judged as a caregiver. The final multiple logistic regression model explained only 21.5% of the variance in sugar intake. This highlights the need for further research on the wider aspects of sugar consumption. Consistency of foods, timing before bedtime, and other methods can offset the high sugar consumption (such as toothbrushing with fluoride toothpaste). It must also be highlighted that the study was undertaken in Columbo, which is just one area of Sri Lanka, which may limit its generalizability. However, the response rate of the survey population was very high.

This is the first study to assess sugar intake in Sri Lanka and is focused on sugar consumption among preschoolers. Similar studies are rare in the available literature, with the majority of published studies from other countries examining sugar intake across a variety of age groups, methodologies, and the type of sugar they refer to, which limits meaningful comparisons and analysis. Most published studies have assessed the intake of added sugar. Although the current study focused on free sugar intake, most food and beverage items in the FFQ were processed foods that contained only added sugars. The free sugar intake calculated in the present study can be considered comparable to the added sugar intake.

To obtain a sense of Sri Lankan sugar consumption, national sugar availability per capita was the only figure available, at 82.19 g/person/day in 2016 [30], which approximately resembles the findings of our study at 79 g/day.

Finnish dietary surveys conducted from to 1998-2003 found that dietary sucrose consumption among 4-year-olds was 53 g/day [31]. Our study population consumed more free sugar than that (79 g/day). These findings are consistent with the findings of a 2020 multicenter European study of free sugar intake among children aged 2-9-years, which identified consumption at 81 g/day for boys and 77 g/day for girls [32].

During the period 2005 to 2008, the National Health and Nutrition Examination Survey of the United States found that on average, preschool-aged boys consumed 13.5% of their calories from added sugars, whereas young girls consumed 13.1% [33]. An analysis of the added sugar intake among 4-13-year-old children in China, Mexico and the USA, conducted in 2018, found that added sugar intake comprised 3% in China, 13% in Mexico and 18% in the USA of total energy intake [34], while we found this measure to be 21.1% for our Sri Lankan preschool population, which is significantly higher than most other contexts in the literature. According to the current study, bakery products, including biscuits, contributed the most to total sugar intake, similar to a study among Chinese children that found that rolls, buns, cakes, and pies contributed most to the added sugar intake. By contrast in two Western countries, Mexico and the United States, studies report that the highest sugar intake is from sugar-sweetened beverages [34], with a European multi-centre study finding that fruit juices accounts for more free sugar intake, followed by soft drinks and dairy products [32].

Regular availability of a particular food group at home was associated with a higher sugar intake for that food group (Table 4). The findings were similar to those of Patrick and Nicklas (2005) [35], suggesting that children mostly eat what they can get their hands on. A second important finding was that those who had fruits available regularly consumed less sugar than did those who did not. An analogous effect of increased fruit and vegetable intake on sugar intake was demonstrated in earlier studies [36–38]. Therefore, public health messages should focus on encouraging parents to make healthier alternatives to sugary foods and drinks available to their children at home.

One of the objectives of this study was to compare the level of sugar intake among the study population and the WHO guidelines on free sugar intake [13]. In the current study population, majority of children exceeded the recommended free sugar intake, 82.7% (*n* = 672) consumed free sugar exceeding 10% of total daily energy intake, whereas 96.3% (*n* = 783) consumed more than 5% of their daily calories through free sugars.

### Factors associated with the pattern of sugar intake

Local health authorities are seeking methods to curb sugar consumption. Sustainable behavioural change can be achieved by identifying and addressing the underlying determinants of high sugar intake. This study identified a few of these determinants.

The final model of the multivariate analysis retained only six variables: ethnicity, maternal education level, presence of school-going siblings, eating while returning from preschool, eating while watching TV, and dental clinic attendance (Table 4).

- *Ethnicity:* The sugar intake of Sinhala children is lower than that of children from other ethnic groups, mainly Tamils or Moors. Several studies have shown the variation in sugar intake among different ethnicities [35, 39].
- *Maternal education level:* This is a more important factor in determining child-related behaviours. Increased maternal education leads to a reduction in children’s sugar intake. Several studies support this finding [39, 40].
- *Presence of school-going siblings:* Associated with high sugar intake. There is no apparent reason for this association – although peer pressure, and the more limited availability and capacity of parents to entertain younger children while also looking after school-age children, may be a factor.
- *Eating habits:* It was found that eating at the time of returning from preschool or watching television was associated with high sugar consumption. Children are more likely to consume sugary foods and beverages on these occasions. These two factors together explain a 12.6% variation in sugar intake, which was also proven in previous studies [40, 41]. These are essential aspects to highlight when instructing caregivers on sugar-reducing techniques, and caregivers should be encouraged to seek healthier alternatives.
- *Dental clinic attendance:* This also showed a negative association with sugar intake and a negative β coefficient. This may be due to their advice in dental clinics, which makes them more concerned about their oral health.

## Conclusion

Compared with children in most other countries, the current population consumes egregiously more free sugar, and the majority of children surpass the WHO-recommended safe free sugar intake levels. Therefore, a robust health promotion programme is needed for young children in Sri Lanka. Public awareness should be created about the levels of sugar that are safe to consume. Target areas and intervention points identified by the study are the available of sugar-containing products in the home, and the time after returning from preschool, especially when children turn to watching TV at this point. The availability of bakery products in Sri Lanka for this population and more healthy alternatives to this, would also be an appropriate intervention target to reduce the relatively high and potentially deleterious levels of sugar consumption in this population.

## Data Availability

The datasets that support the findings of the study are available from the corresponding author on reasonable request.

## Abbreviations

95% CI: 95% Confidence Interval
FFQ: Food Frequency Questionnaire
24hDR: 24 Hour Dietary Record
IQR: Inter-Quartile Range
NCD: Noncommunicable diseases
P: probability value
WHO: World Health Organization
